# Depressive Symptoms and Its Associated Factors in 13-Year-Old Urban Adolescents

**DOI:** 10.3390/ijerph10105026

**Published:** 2013-10-14

**Authors:** Cláudia Bulhões, Elisabete Ramos, Jutta Lindert, Sónia Dias, Henrique Barros

**Affiliations:** 1 Department of Clinical Epidemiology, Predictive Medicine and Public Health, University of Porto Medical School, Porto 4200-319, Portugal; E-Mails: eliramos@med.up.pt (E.R.); hbarros@med.up.pt (H.B.); 2 Institute of Public Health, University of Porto (ISPUP), Porto 4050-600, Portugal; 3 Department of Public Health, University of Ludwigsburg, Ludwigsburg 71638, Germany; E-Mail: mail@jlindert.de; 4 International Public Health and Biostatistics Unit & CMDT, Instituto de Higiene e Medicina Tropical, Universidade Nova de Lisboa, Lisbon 1349-008, Portugal; E-Mail: smfdias@yahoo.com

**Keywords:** adolescent, depression, epidemiology, health behavior

## Abstract

The available estimates reveal that 20–50% of adolescents report depressive symptoms, being one of the most prevalent health problems in adolescence. The aim of this study was to assess the prevalence of depressive symptoms in a community sample of 13-year-old adolescents and identify associated features. Thirteen year-old adolescents attending private and public schools in Porto (n = 1,988, 52.2% females) were evaluated from October 2003 to June 2004 and completed a questionnaire including health behaviors and the Beck Depression Inventory II. A questionnaire on parents’ socio-demographics and clinical characteristics was sent home. Data were analyzed separately by sex. Odds ratios (ORs) and 95% confidence intervals (CIs) were estimated using logistic regression. The prevalence of depressive symptoms was 18.8% in girls and 7.6% in boys (*p* < 0.001). Boys with a family history of depression and girls with smoking habits had a significantly increased risk of depressive symptoms (OR = 2.18, 95%CI 1.00–4.71; OR = 2.34, 95%CI 1.46–3.76). Menarche at an early age significantly increased the risk of depressive symptoms. The characteristics most strongly associated with depressive symptoms were family history of depression among boys, tobacco consumption and an early age at menarche among girls. The high prevalence of depressive symptoms early in adolescence calls for the awareness of public health professionals.

## 1. Introduction

Depression is one of the most common psychiatric disorders of adolescence [1]. In European population samples, 4% of 12–17-year-olds and 9% of 18-year-olds were classified as suffering from depression [2]. Additionally, community surveys based on self-reported symptom scales have revealed that between 20 and 50% of adolescents exceed conventionally established cut-off points for clinically significant depression [3,4]. 

Depressive symptoms, though not a sufficient criteria for the clinical diagnosis of depressive disorder, seem to be quite stable throughout adolescence, with individuals who experience depressive symptoms earlier in adolescence being more likely to continue reporting depressive symptoms later [5,6]. Depressive symptoms were often attributed to the normal stress of adolescence, however, it is increasingly recognized that depression often begins in this life period [4,7,8]. In present time cultures adolescence may be seen as a sensitive period, when youth begin to experience a desire for intimacy and an increase of both social and academic responsibilities, being a crucial period for mental health problems as cognitions and emotions may develop in a non-efficient way [4]. Depression during adolescence is predictive of both depressive symptoms and depressive disorders in adulthood. In a prospective study [7], in which a birth cohort of individuals (n = 1,037) was followed for 26 years, 75% of adults at age 26 who met criteria for major depression, had already presented a depressive disorder in childhood or adolescence and only 25% had experienced the onset of depression in adulthood. Similar results were reported in another prospective community study of 274 adolescents, in which about one-fourth of formerly depressed adolescents experienced subsequent pure major depressive disorder, one-fourth experienced comorbid major depressive disorder, and one fourth remained free from depression recurrence, but experienced a non-mood disorder [8]. 

Suicide is the most devastating outcome of depressive symptoms during adolescence and is the second cause of death in this age group in Western Europe [9]. Depressive symptoms may also affect the process of socialization, family relationships and school performance. Adolescents presenting depressive symptoms are also at increased risk for alcohol or substance abuse and antisocial behaviors [10,11,12].

There is a gender difference in the prevalence of depressive symptoms during adolescence but not at childhood; nevertheless, the specific age when this difference begins is controversial [1,13,14,15,16]. The emergence of this gender difference in depression rates has been found to vary from as early as 10 to 14 years of age [14,17] to 15 to 19 years of age [18]. The individual and family factors associated with depressive symptoms early in adolescence, in samples of the general population, are also controversial, with most studies including older adolescents, and the specific weighting of these factors is not yet known [5,19,20,21,22,23].

The analysis of a large population based sample of adolescents at the same age is critical to understand this issue and the gender differences. This information can also contribute to the development of effective interventions to minimize impairments associated to depressive symptoms during adolescence and to prevent major depression. The aim of this study was to assess the prevalence of depressive symptoms in school students at 13 years of age and secondly, to identify individual and family factors associated with adolescents’ depressive symptoms.

## 2. Methods

### 2.1. Subjects

Full details of the Epidemiological Health Investigation of Teenagers in Porto (EPITeen) have been described elsewhere [24]. In brief, participants were evaluated from October 2003 to June 2004, during the assembling of the cohort of urban adolescents born in 1990. Written informed consent was obtained both from adolescents and their parents or legal guardians. The study was approved by the ethics committee of the São João University Hospital.

We identified 2,787 eligible adolescents (2,126 in public and 661 in private schools). Forty-four (1.6%) could not be reached (missing classes during the study period) and 582 (20.9%) did not return signed consent forms and were considered refusals. This resulted in a 77.5% overall proportion of participation, similar in public (77.9%) and private schools (77.0%; *p* = 0.71), with 2,160 students providing information for at least part of the proposed assessment. For the present analysis, 172 (6.2%) adolescents were excluded because of missing Beck Depression Inventory II (BDI-II) information. The adolescents who completed the BDI-II were significantly different from the remaining participants regarding some characteristics. Their parents reported a higher level of formal education (28.7% *vs*. 17.2%; *p* = 0.016, among boys) and not having history of depression (55.6% *vs.* 33.3%; *p* = 0.001, among girls). A lower proportion of girls were pre-menarche (9.3% *vs.* 13.6%) and sleeping 8.5 h or less was also more frequent among girls who completed BDI-II (31.8% *vs.* 23.8%; *p* < 0.001). Additionally, boys who completed the BDI-II less frequently ever smoked (19.0% *vs.* 38.5%; *p* = 0.013) and alcohol consumption was more frequent among girls who completed BDI-II (55.4% *vs.* 25.0%; *p* = 0.015). No significant differences were found regarding the other variables analysed in this study. The final sample includes 1,988 students (1,037 girls (52.2%) and 951 boys (47.8%)).

### 2.2. Adolescents’ Information

The information was obtained using two self-administered questionnaires, created by the research team, and through a physical examination. One questionnaire was completed at school before physical examination, during the field team visit, and another was completed at home. 

The school questionnaire comprised information about health-related behaviors, namely tobacco and alcohol consumption, and physical activity. Concerning tobacco and alcohol, adolescents were classified in non-smokers/drinkers if they had never used; and as smokers/drinkers if they had experimented or if they smoke/drink regardless of the frequency and quantity. Relating to physical activity, adolescents were asked if they practice any activity, excluding school activities, and how many times per week. 

#### Depressive Symptoms

To assess depressive symptoms, the Beck Depression Inventory II (BDI-II) was employed. It is a self-report instrument for measuring depression in adults and adolescents aged 13 years and older. It consists of 21 items and each item is rated on a 4-point scale ranging from 0 to 3 [25]. Participants were asked to endorse the most characteristic statements covering the time frame of the most recent two weeks, including that day. Responses are summed, yielding a range of scores from 0 to 63, with higher scores indicating more depressive symptoms. The BDI-II was previously validated for the Portuguese adolescents [26]. The cut-off used to define adolescents presenting depressive problems was >13 [26]. Anthropometrics were obtained at school with the subject in light indoor clothes and no shoes. Body mass index (BMI) was classified according to the age- and sex-specific BMI percentiles, elaborated by the United States Centers for Disease Control and Prevention, as overweight (BMI between the 85th and the 95th percentile) and obesity (BMI above the 95th percentile) [27]. Age at menarche was self-reported and considered an indicator of pubertal development. 

### 2.3. Parents’ Information

Parents were asked to complete a questionnaire, at home, providing information about them and the adolescent on social, demographic and health related items. Adolescents were classified taking into account the parent with a higher level of education and this information was used as a proxy for socioeconomic status. In order to evaluate family history of depression, each parent was asked for a previous clinical diagnosis of depression, made by a health care provider. The possible answers were *yes*, *no* and *do not know*. Subsequently, the adolescents were stratified as not exposed to parental depression when both parents reported not having a previous diagnosis of depression and exposure to parental depression was considered when at least one parent answered *yes*. It was also considered the option *do* *not know* when both parents reported not knowing about a previous diagnosis or if one parent reported not having this diagnosis and the information of the other parent was *not know* or was missing. 

### 2.4. Data Analyses

Separate analyses were performed for boys and girls, firstly using the BDI-II score as a continuous variable, measuring median and inter-quartile ranges, and secondly assessing the prevalence of depressive symptoms (BDI-II > 13), according to adolescents’ characteristics. Data was compared with Mann Whitney, Kruskal-Wallis and Chi-Square tests. Odds ratios and 95% confidence interval (95%CI) were estimated using unconditional logistic regression. *P*-values less than 0.05 were considered statistically significant. Statistical analyses were performed using SPSS version 17.0 (SPSS Inc., Chicago, IL, USA).

## 3. Results

The overall prevalence of depressive symptoms (BDI-II score > 13) was 13.4% in the total sample, 18.8% in girls and 7.6% in boys (*p* < 0.001). The median score (P25–P75) of the depressive symptoms was 6.01 (3.00–11.00) and 3.00 (1.01–6.99) among females and males, respectively (*p* < 0.001). 

Boys’ and girls’ median BDI-II scores according to family and individual characteristics and behaviors are listed in Table 1. Both boys and girls who reported alcohol consumption and girls living without both parents, with family history of depression, with an early age at menarche, sleeping 8.5 h or less and with history of tobacco smoke presented a significantly higher score of depressive symptoms, when compared to their peers. 

**Table 1:** Beck Depression Inventory scores (median and P25–P75) according adolescents’ characteristics, by sex (Porto, Portugal 2003–2004) (n = 1,988).

	BDI-II				
	Girls (n = 1,037)			Boys (n = 951)	
Characteristics	Median (P25–P75)	*p-value* ^‡^		Median (P25–P75)	*p-value* ^‡^
*Family characteristics*					
**Parents’ education, year**					
0–6	6.99 (3.00–12.24)	0.163		3.00 (1.01–6.01)	0.143
7–9	5.12 (2.00–10.00)			3.00 (0.00–6.01)	
10–12	6.01 (2.00–11.00)			3.08 (1.01–6.01)	
≥13	6.01 (3.00–10.00)			3.16 (1.01–7.17)	
**Household**					
Both parents	6.01 (2.00–10.00)	**0.004**		3.00 (1.01–6.01)	0.618
Mother/father/other	6.99 (3.99–11.99)			3.00 (1.01–7.35)	
**Parents’ depression**					
None	5.00 (2.00–10.00)	**0.001**		3.00 (1.01–6.01)	0.054
At least 1 parent	6.99 (3.00–11.99)			3.99 (1.01–8.00)	
Do not know	6.01 (2.00–9.01)			2.05 (0.00–6.01)	
*Individual characteristics*					
**Age at menarche, year**					
≤10	6.99 (3.00–15.75)	**0.010**			
11	6.99 (3.00–11.99)				
12	6.01 (2.00–11.55)				
≥13	5.00 (2.00–9.01)				
Pre-menarche	5.00 (2.00–8.04)				
**BMI ** ^†^					
<p85	6.01 (2.00–11.00)	0.228		3.00 (1.01–6.01)	0.813
≥p85 & <p95	6.99 (3.00–10.00)			3.50 (1.01–6.99)	
≥p95	6.01 (3.00–13.00)			3.00 (1.01–6.99)	
*Behaviours*					
**Sleeping duration, h**					
8.5–9.5	5.63 (2.00–10.00)	**0.012**		3.00 (1.01–6.01)	0.956
≤8.5	6.99 (3.00–11.99)			3.00 (1.01–6.01)	
≥9.5	6.01 (3.00–11.00)			3.00 (1.01–6.01)	
**Smoking**					
Never	5.00 (2.00–9.45)	**<0.001**		3.00 (1.01–6.01)	0.072
Ever	8.40 (3.99–15.75)			3.00 (1.01–8.00)	
**Drinking alcohol**					
Never	6.01 (2.00–10.00)	**0.002**		3.00 (1.01–6.01)	**0.038**
Ever	6.30 (3.00–11.99)			3.99 (1.01–6.99)	
**Physical exercise**					
Never	6.30 (3.00–11.99)	0.152		3.00 (1.01–6.99)	0.127
≤1 time/week	5.00 (3.00–9.01)			3.00 (1.01–6.01)	
2–3 times/week	6.01 (2.00–10.00)			3.00 (1.01–6.01)	
≥4 times/week	4.49 (2.00–6.99)			3.99 (1.01–8.00)	

Abbreviations: BDI-II, Beck Depression Inventory, Second Edition; BMI, body mass index (calculated as weight in kilograms divided by height in meters squared). ^†^ BMI was classified according to the age- and sex-specific BMI percentiles, elaborated by the United States Centers for Disease Control and Prevention. ^‡^ Mann Withney or Kruskal-Wallis tests.

Adolescents with a history of tobacco consumption presented a higher prevalence of depressive symptoms (Table 2). The relationship between living with both parents and depressive symptoms approached significance for boys, with boys living with both parents less likely to exceed the BDI-II cutoff score. The relationship between living with parents and depressive symptoms was not significant for girls. The relationship between parent history of depression and depressive symptoms was not significant for either boys or girls (Table 2). 

**Table 2:** Prevalence of depressive symptoms (BDI-II > 13) according to adolescents’ characteristics for girls and boys (Porto, Portugal 2003–2004) (n = 1,988).

	Girls (n = 1,037) n (%)				Boys (n = 951) n (%)		
Characteristics	*BDI-II ≤ 13*	*BDI-II > 13*	*p*-value ^‡^		*BDI-II ≤ 13*	*BDI-II > 13*	*p*-value ^‡^
*Family characteristics*							
**Parents’ education, year**							
0–6	226 (27.6)	63 (34.4)	0.199		191 (22.7)	24 (38.7)	**0.029**
7–9	168 (20.5)	40 (21.9)			174 (20.6)	8 (12.9)	
10–12	211 (25.8)	42 (23.0)			235 (27.9)	13 (21.0)	
≥13	213 (26.0)	38 (20.8)			243 (28.8)	17 (27.4)	
**Household**							
Both parents	632 (75.1)	140 (72.2)	0.390		696 (79.3)	50 (69.4)	0.051
Mother/father/other	209 (24.9)	54 (27.8)			182 (20.7)	22 (30.6)	
**Parents’ depression**							
None	291 (56.7)	54 (50.5)	0.256		297 (57.4)	13 (41.9)	0.146
At least 1 parent	164 (32.0)	43 (40.2)			163 (31.5)	15 (48.4)	
Do not know	58 (11.3)	10 (9.3)			57 (11.0)	3 (9.7)	
*Individual characteristics*							
**Age at menarche, year**							
≤10	65 (8.1)	26 (14.2)	**0.026**				
11	178 (22.2)	45 (24.6)					
12	281 (35.1)	66 (36.1)					
≥13	148 (18.5)	28 (15.3)					
Pre-menarche	128 (16.0)	18 (9.8)					
**BMI** ^†^							
<p85	622 (74.4)	147 (76.2)	0.338		623 (71.5)	56 (78.9)	0.414
≥p85 & < p95	140 (16.7)	25 (13.0)			147 (16.9)	9 (12.7)	
≥p95	74 (8.9)	21 (10.9)			101 (11.6)	6 (8.5)	
**Sleeping duration, h**							
8.5–9.5	305 (40.0)	61 (34.5)	0.298		311 (41.4)	15 (31.2)	0.367
≥9.5	223 (29.2)	52 (29.4)			243 (32.4)	19 (39.6)	
≤8.5	235 (30.8)	64 (36.2)			197 (26.2)	14 (29.2)	
*Behaviours*							
**Smoking**							
Never	640 (76.8)	111 (57.5)	**<0.001**		697 (81.8)	51 (71.8)	**0.039**
Ever	193 (23.2)	82 (42.5)			155 (18.2)	20 (28.2)	
**Drinking alcohol**							
Never	385 (46.2)	71 (37.6)	**0.032**		413 (48.0)	31 (44.3)	0.553
Ever	449 (53.8)	118 (62.4)			448 (52.0)	39 (55.7)	
**Physical exercise**							
Never	490 (59.3)	125 (65.8)	0.240		335 (38.7)	30 (44.1)	**0.028**
≤1 time/week	120 (14.5)	21 (11.1)			117 (13.5)	5 (7.4)	
2–3 times/week	171 (20.7)	38 (20.0)			301 (34.8)	17 (25.0)	
≥4 times/week	46 (5.6)	6 (3.2)			113 (13.0)	16 (23.5)	

Abbreviations: BDI-II, Beck Depression Inventory, Second Edition; BMI, body mass index (calculated as weight in kilograms divided by height in meters squared). ^†^ BMI was classified according to the age- and sex-specific BMI percentiles, elaborated by the United States Centers for Disease Control and Prevention. ^‡^ Chi-Square test.

An early age at menarche and alcohol consumption was associated with a higher prevalence of depressive symptoms among girls (Table 2). Boys with less educated parents and boys who revealed not doing physical exercise or doing physical activity more than four times/week, had higher prevalence of depressive symptoms (Table 2). 

**Table 3:** Crude and adjusted odds ratios for depressive symptoms (BDI-II > 13), according to boys’ and girls’ characteristics (Porto, Portugal 2003-2004) (n = 1,988).

		Girls			Boys	
		OR (95%CI)			OR (95%CI)	
Characteristics		Crude		Adjusted ^‡^	Crude	Adjusted ^‡^
*Family characteristics*						
**Parents’ education, year**						
0–6	1		1		1	1
7–9	0.85 (0.55–1.33)		0.89 (0.48–1.66)		0.37 (0.16–0.84)	0.27 (0.07–0.99)
10–12	0.71 (0.46–1.10)		0.71 (0.40–1.26)		0.44 (0.22–0.89)	0.33 (0.12–0.94)
≥13	0.64 (0.41–0.99)		0.79 (0.45–1.39)		0.56 (0.29–1.07)	0.60 (0.25–1.46)
**Household**						
Both parents	1		1		1	1
Mother/father/other	1.17 (0.82–1.66)		1.13 (0.66–1.94)		1.68 (0.99–2.85)	1.09 (0.39–3.04)
**Parents’ depression**						
None	1		1		1	1
At least 1 parent	1.41 (0.91–2.20)		1.42 (0.90–2.22)		2.10 (0.98–4.53)	2.18 (1.00–4.74)
Do not know	0.93 (0.45–1.93)		0.88 (0.42–1.85)		1.20 (0.33–4.36)	1.00 (0.27–3.71)
*Individual characteristics*						
**Age at menarche, year**						
Pre-menarche	1		1			
≥13	1.35 (0.71–2.55)		2.89 (1.02–8.22)			
12	1.67 (0.95–2.93)		3.59 (1.36–9.51)			
11	1.80 (1.00–3.25)		4.12 (1.51–11.21)			
≤10	2.84 (1.45–5.56)		6.07 (2.00–18.46)			
**BMI ^†^**						
<p85	1		1		1	1
≥p85 & <p95	0.76 (0.48–1.20)		0.66 (0.35–1.24)		0.68 (0.33–1.41)	0.66 (0.22–1.97)
≥p95	1.20 (0.72–2.01)		0.85 (0.38–1.90)		0.66 (0.28–1.57)	0.49 (0.11–2.15)
*Behaviours*						
**Sleeping duration, h**						
8.5–9.5	1		1		1	1
≥9.5	1.17 (0.78–1.75)		0.94 (0.55–1.60)		1.62 (0.81–3.26)	2.36 (0.89–6.26)
≤8.5	1.36 (0.92–2.01)		1.11 (0.67–1.84)		1.47 (0.70–3.12)	1.74 (0.63–4.78)
**Smoking**						
Never	1		1		1	1
Ever	2.45 (1.77–3.40)		2.57 (1.65–4.01)		1.76 (1.02–3.04)	1.43 (0.61–3.37)
**Drinking alcohol**						
Never	1		1		1	1
Ever	1.43 (1.03–1.97)		1.52 (0.97–2.37)		1.16 (0.71–1.89)	1.35 (0.62–2.92)
**Physical exercise**						
Never	1		1		1	1
≤1 time/week	0.69 (0.42–1.14)		0.61 (0.31–1.22)		0.48 (0.18–1.26)	0.52 (0.11–2.50)
2–3 times/week	0.87 (0.58–1.30)		0.93 (0.54–1.62)		0.63 (0.34–1.17)	1.00 (0.36–2.79)
≥4 times/week	0.51 (0.21–1.22)		0.67 (0.22–2.02)		1.58 (0.83–3.00)	2.59 (0.89–7.54)

Abbreviations: BDI-II, Beck Depression Inventory, Second Edition; BMI, body mass index (calculated as weight in kilograms divided by height in meters squared); CI, confidence interval. ^†^ BMI was classified according to the age- and sex- specific BMI percentiles, elaborated by the United States Centers for Disease Control and Prevention. ^‡^ OR estimates adjusted for parent’s education and parent’s depression.

After adjusting for parent’s education and family history of depression, the odds of depressive symptoms was higher among girls with an early age at menarche and with history of tobacco consumption (Table 3). Girls who reported alcohol consumption also had a higher risk of depressive symptoms, although this result was not statistically significant (Table 3). Having less educated parents and having family history of depression were associated with depressive symptoms among boys (Table 3).

## 4. Discussion

The prevalence of depressive symptoms found in this study is in line with previous estimates [19,20,21,22,23]. Although preceding research has shown a similar frequency of depressive symptoms among adolescents, most studies included older adolescents, mainly from high school and college [19,20,21,22,23]. As it is known that the rates of depressive symptoms increase during adolescence [3,10,13], the prevalence found in this study among young adolescents might predict a higher impact of depression later in life than expected, with a higher prevalence of depression in adulthood.

The prevalence of depressive symptoms was more than twice as common in girls as in boys. This large difference found by this time among 13-year-old adolescents suggests that sex differences in depression may emerge earlier in life, and not only at 13 years of age, as previous research has stated [1,14,15]. Pubertal development can partially explain the sex differences in the prevalence of depressive symptoms as the emergence of a female excess of symptoms during adolescence is probably due to some combination of age-related changes in biological and cognitive functioning or social circunstances [4,14,28,29]. In this study, because there were no conditions at school to assure the privacy needed to evaluate adolescents’ pubertal development according to Tanner criteria, timing of menarche was used as an indicator of pubertal status. Although this option did not allow to compare boys and girls, girls with an early age at menarche presented a higher proportion of depressive symptoms. These findings, although presenting large confidential intervals due to the reduced sample size of some categories, minimizing the accuracy of the estimates, are not inconsistent with the social model for the impact of puberty development on depression. The change in social expectations and reactions of others to the adolescent's puberty development are more challenging for an individual entering the process without the support from peers in similar situation [30].

The relationship between low socioeconomic status, measured using parental education as a proxy, and the presence of depressive symptoms, with significant results only among boys, is in line with previous studies [31]. This can be explained by differences in coping styles and other interpersonal skills, such as communication, and differences in access to material goods and services [32].

As shown in previous studies, it was also found that adolescents whose parents reported receiving a diagnosis of depression at some point in their lives were more likely to report depressive symptoms [4,15,28]. This result underlines that depressive symptoms among these adolescents may not be a normal or temporary stage of adolescent’s development since it is known that having a parent with a history of major depression is one of the strongest predictors of depression in youth [4]. However it is not possible to conclude if this relationship is due to genetic factors and heritability or to a shared common family environment. It can even stress the importance of cultural factors running in families and making the expression of feelings and its communication more socially acceptable and following a common pattern. Culture affects parenting strategies and styles, and the development of emotions and self-concept, key components of adolescents’ lives, affecting the adolescents’ perceptions of themselves [33]. Furthermore, culture is related with the individuals sensitivity to stressful life events [34]. Family environmental factors, also linked with cultural factors, like lack of family closeness, poor communication, absence of supportive relationships, parental rejection and little emotional warmth have all been found to be prevalent in families with depressed parents and are associated with an increased risk of emotional and behavioural problems in offspring [35,36]. Evidence from research on twins suggests that depressive symptoms are heritable starting in adolescence (after age 11) and continuing throughout adulthood, whereas family environment, but no genetic factors, is linked with depression in childhood (before age 11) [4]. In the present research age at symptoms presentation was not asked and thus it is not possible to speculate on the relative importance of the different aetiologies. These results go on-line with previous described associations besides the often recognized limitations of self-reported data regarding family history of depression’s variable. This study showed that girls with history of tobacco consumption had higher levels of depressive symptoms, in accordance with other studies [3,5,37,38]. Like previous research stated, there was a stronger link between depression and substance problems among females [10,39,40] that can be related to girls being more vulnerable to social influences and to consequences of substance use than boys [40]. 

No meaningful association between depressive symptoms and physical exercise was found, in opposition to other studies that support an association between more frequent physical activity and lower levels of depression among adolescents [41,42,43]. There may be a higher probability of finding this inverse relationship in clinical samples than in population-based samples since the effects of physical activity are likely to be greater in adolescents who have poor mental health at baseline [43,44]. In addition, although physical activity may enhance psychological well-being, it is possible that the predominant psychological environment and social interactions inherent in such settings will also be crucial [43].

The strength of this study relies on its population-base and the fact that it was conducted within a large sample of adolescents at an early phase of adolescence. As school education is compulsory in Portugal until 15 years of age all eligible adolescents were accessible at school. Nevertheless, 6.2% of the adolescents did not complete the BDI and those who completed this inventory belonged to families with higher educational level, showed a lower proportion of girls in pre-menarche and of boys with smoking habits and a higher proportion of girls consuming alcohol. According to known associations some of these differences could result in an artificially higher prevalence of depressive symptoms whereas others could cause the opposite. Once the number of adolescents who did not complete the BDI was small it is not expectable that these differences could affect the prevalence found and biase the associated measures. 

Other limitations need to be addressed. As in previous studies [13], our findings might be hampered by the relatively small number of boys presenting depressive symptoms. Secondly, though depressive symptoms exist as a continuum in the population, it was not possible to utilize these as continuous data due to the highly positively skewed distribution of scores. However, a binary outcome variable (depressed *vs*. not depressed) is expected to be clinically more relevant. Thirdly, although the measure of depression used is one of the most commonly used scales for detecting possible depression in normal population, that closely parallels Diagnostic and Statistical Manual IV (DSM-IV) symptoms of depression and proved to be a valid method among adolescents, we had no formal diagnosis of depression, and this self-report measure for screening depression does not necessarily refer to depression of clinical relevance [45]. Nevertheless, our objective was not to measure the prevalence of clinical depression among adolescents but to assess depressive symptoms in this population since it is now understood that these symptoms represent a significant public health concern contributing to illness and disability [28]. 

## 5. Conclusions

Regardless the limitations, this study identifies factors related to depressive symptoms in a large sample comprising girls and boys at 13 years of age, from a nonclinical population. We consider that the prevalence of depressive symptoms found in our study (19% in girls and 8% in boys) calls for the awareness of public health professionals and politicians and justifies being alert to adolescents’ own indications of distress, already in the early phase of adolescence. Besides this, as the course of adolescents’ depressive symptoms has been noticed to resemble that of major depression, using self-reported screening questionnaires may help identifying these adolescents in order to guide them to appropriate helping interventions. 

## References

[1] Costello E.J., Mustillo S., Erkanli A., Keeler G., Angold A. (2003). Prevalence and development of psychiatric disorders in childhood and adolescence. Arch. Gen. Psychiatry.

[2] World Health Organization (2005). The Health of Children and Adolescents in Europe. http://internazionali.ulss20.verona.it/docs/projects/adrisk/eurosafe03.pdf.

[3] Kessler R.C., Avenevoli S., Ries Merikangas K. (2001). Mood disorders in children and adolescents: An epidemiologic perspective. Biol. Psychiatry.

[4] Hankin B.L. (2006). Adolescent depression: Description, causes, and interventions. Epilepsy Behav..

[5] Haavisto A., Sourander A., Multimaki P., Parkkola K., Santalahti P., Helenius H., Nikolakaros G., Kumpulainen K., Moilanen I., Piha J. (2004). Factors associated with depressive symptoms among 18-year-old boys: A prospective 10-year follow-up study. J. Affect. Disord..

[6] Pine D.S., Cohen E., Cohen P., Brook J. (1999). Adolescent depressive symptoms as predictors of adult depression: Moodiness or mood disorder?. Am. J. Psychiatry.

[7] Kim-Cohen J., Caspi A., Moffitt T.E., Harrington H., Milne B.J., Poulton R. (2003). Prior juvenile diagnoses in adults with mental disorder: Developmental follow-back of a prospective-longitudinal cohort. Arch. Gen. Psychiatry.

[8] Lewinsohn P.M., Rohde P., Seeley J.R., Klein D.N., Gotlib I.H. (2000). Natural course of adolescent major depressive disorder in a community sample: Predictors of recurrence in young adults. Am. J. Psychiatry.

[9] Patton G.C., Coffey C., Sawyer S.M., Viner R.M., Haller D.M., Bose K., Vos T., Ferguson J., Mathers C.D. (2009). Global patterns of mortality in young people: A systematic analysis of population health data. Lancet.

[10] Saluja G., Iachan R., Scheidt P.C., Overpeck M.D., Sun W., Giedd J.N. (2004). Prevalence of and risk factors for depressive symptoms among young adolescents. Arch. Pediatr. Adolesc. Med..

[11] Bhatia S.K., Bhatia S.C. (2007). Childhood and adolescent depression. Am. Fam. Physician.

[12] Zuckerbrot R.A., Jensen P.S. (2006). Improving recognition of adolescent depression in primary care. Arch. Pediatr. Adolesc. Med..

[13] Van Lang N.D., Ferdinand R.F., Verhulst F.C. (2007). Predictors of future depression in early and late adolescence. J. Affect. Disord..

[14] Angold A., Costello E.J., Worthman C.M. (1998). Puberty and depression: The roles of age, pubertal status and pubertal timing. Psychol. Med..

[15] Weissman M.M., Warner V., Wickramaratne P., Moreau D., Olfson M. (1997). Offspring of depressed parents. 10 Years later. Arch. Gen. Psychiatry.

[16] De Boo G.M., Spiering M. (2010). Pre-adolescent gender differences in associations between temperament, coping, and mood. Clin. Psychol. Psychother..

[17] Kessler R.C., McGonagle K.A., Swartz M., Blazer D.G., Nelson C.B. (1993). Sex and depression in the National Comorbidity Survey. I: Lifetime prevalence, chronicity and recurrence. J. Affect. Disord..

[18] Burke K.C., Burke J.D., Regier D.A., Rae D.S. (1990). Age at onset of selected mental disorders in five community populations. Arch. Gen. Psychiatry.

[19] Frojd S.A., Nissinen E.S., Pelkonen M.U., Marttunen M.J., Koivisto A.M., Kaltiala-Heino R. (2008). Depression and school performance in middle adolescent boys and girls. J. Adolesc..

[20] Purvis D., Robinson E., Merry S., Watson P. (2006). Acne, anxiety, depression and suicide in teenagers: A cross-sectional survey of New Zealand secondary school students. J. Paediatr. Child. Health.

[21] Escriba Quijada R., Maestre Montoya C., Amores Laserna P., Pastor Toledo A., Miralles Marco E., Escobar Rabadan F. (2005). Depression prevalence in adolescents. Actas Esp. Psiquiatr..

[22] Rethelyi J.M., Berghammer R., Ittzes A., Szumska I., Purebl G., Csoboth C. (2004). Comorbidity of pain problems and depressive symptoms in young women: Results from a cross-sectional survey among women aged 15–24 in Hungary. Eur. J. Pain.

[23] Haavet O.R., Straand J., Saugstad O.D., Grunfeld B. (2004). Illness and exposure to negative life experiences in adolescence: Two sides of the same coin? A study of 15-year-olds in Oslo, Norway. Acta Paediatr..

[24] Ramos E., Barros H. (2007). Family and school determinants of overweight in 13-year-old Portuguese adolescents. Acta Paediatr..

[25] Beck A.T., Steer R.A., Brown G.K. (1996). Manual for the Beck Depression Inventory-II.

[26] Coelho R., Martins A., Barros H. (2002). Clinical profiles relating gender and depressive symptoms among adolescents ascertained by the Beck Depression Inventory II. Eur. Psychiatry.

[27] Kuczmarski R.J., Ogden C.L., Guo S.S., Grummer-Strawn L.M., Flegal K.M., Mei Z., Wei R., Curtin L.R., Roche A.F., Johnson C.L. 2000 CDC Growth Charts for the United States: Methods and Development. http://www.cdc.gov/growthcharts/2000growthchart-us.pdf.

[28] Garber J. (2006). Depression in children and adolescents: Linking risk research and prevention. Am. J. Prev. Med..

[29] Chaplin T.M., Gillham J.E., Seligman M.E. (2009). Gender, anxiety, and depressive symptoms: A longitudinal study of early adolescents. J. Early Adolesc..

[30] Kaltiala-Heino R., Kosunen E., Rimpela M. (2003). Pubertal timing, sexual behaviour and self-reported depression in middle adolescence. J. Adolesc..

[31] Lemstra M., Neudorf C., D’Arcy C., Kunst A., Warren L.M., Bennett N.R. (2008). A systematic review of depressed mood and anxiety by SES in youth aged 10–15 years. Can. J. Public Health.

[32] Goodman E., Slap G.B., Huang B. (2003). The public health impact of socioeconomic status on adolescent depression and obesity. Am. J. Public Health.

[33] Sheeber L., Hops H., Davis B. (2001). Family processes in adolescent depression. Clin. Child Fam. Psychol. Rev..

[34] Rothbart M.K., Ahadi S.A., Evans D.E. (2000). Temperament and personality: Origins and outcomes. J. Personal. Soc. Psychol..

[35] Pilowsky D.J., Wickramaratne P., Nomura Y., Weissman M.M. (2006). Family discord, parental depression, and psychopathology in offspring: 20-Year follow-up. J. Am. Acad. Child Adolesc. Psychiatry.

[36] Meares R. (2000). Priming and projective identification. Bull. Menninger Clin..

[37] Richardson L.P., Katzenellenbogen R. (2005). Childhood and adolescent depression: The role of primary care providers in diagnosis and treatment. Curr. Probl. Pediatr. Adolesc. Health Care.

[38] Chaiton M.O., Cohen J.E., O’Loughlin J., Rehm J. (2009). A systematic review of longitudinal studies on the association between depression and smoking in adolescents. BMC Public Health.

[39] Marmorstein N.R. (2009). Longitudinal associations between alcohol problems and depressive symptoms: Early adolescence through early adulthood. Alcohol Clin. Exp. Res..

[40] Waller M.W., Hallfors D.D., Halpern C.T., Iritani B.J., Ford C.A., Guo G. (2006). Gender differences in associations between depressive symptoms and patterns of substance use and risky sexual behavior among a nationally representative sample of U.S. adolescents. Arch. Womens Ment. Health.

[41] Desha L.N., Ziviani J.M., Nicholson J.M., Martin G., Darnell R.E. (2007). Physical activity and depressive symptoms in American adolescents. J. Sport Exerc. Psychol..

[42] Hong X., Li J., Xu F., Tse L.A., Liang Y., Wang Z., Yu I.T., Griffiths S. (2009). Physical activity inversely associated with the presence of depression among urban adolescents in regional China. BMC Public Health.

[43] Biddle S.J., Asare M. (2011). Physical activity and mental health in children and adolescents: A review of reviews. Br. J. Sports Med..

[44] Johnson C.C., Murray D.M., Elder J.P., Jobe J.B., Dunn A.L., Kubik M., Voorhees C., Schachter K. (2008). Depressive symptoms and physical activity in adolescent girls. Med. Sci. Sports Exerc..

[45] Ambrosini P.J., Metz C., Bianchi M.D., Rabinovich H., Undie A. (1991). Concurrent validity and psychometric properties of the Beck Depression Inventory in outpatient adolescents. J. Am. Acad. Child Adolesc. Psychiatry.

